# Satellite‐Driven Synthesis of Fish Production Dynamics and Carrying Capacity Mechanisms in a High‐Altitude Lake Ecosystem

**DOI:** 10.1002/ece3.72989

**Published:** 2026-02-17

**Authors:** Ban Xuan, Qi Hongfang, Shu Peng, Luo Ying, Ling Feng, Xiao Fei, Li Pengchen, Fu Shengyun, Du Hao

**Affiliations:** ^1^ Hubei Key Laboratory of Environmental and Disaster Monitoring and Assessment Chinese Academy of Sciences Innovation Academy for Precision Measurement Science and Technology Wuhan P. R. China; ^2^ Qinghai Key Laboratory of Breeding and Protection of Naked Carp Qinghai Lake Naked Carp Rescue Center Xining P. R. China; ^3^ State Key Laboratory of Water Resources and Hydropower Engineering Science Wuhan University Wuhan P. R. China; ^4^ Key Laboratory of Freshwater Diversity Protection, Ministry of Agriculture and Rural Affairs, Yangtze River Fisheries Research Institute Chinese Academy of Fishery Sciences Wuhan P. R. China

**Keywords:** fish carrying capacity index, fish potential production, primary production, Qinghai lake, vertically generalised production model

## Abstract

Understanding how limited energy constrains fish populations in fragile high‐altitude lakes is essential for sustainable fisheries management. This study developed a satellite‐based framework that integrated MODIS‐derived chlorophyll *a* data with a vertically generalised production model. This framework was used to map Fish Potential Production (FPP) and establish a novel Fish Carrying Capacity Index (FCCI) for the endemic naked carp ( *Gymnocypris przewalskii* ) in Qinghai Lake, China, from 2002 to 2024. Over the 23‐year period, lake‐wide fish production increased 50‐fold (from 2592 to 127,500 t) alongside an upward trend in FPP driven by primary production. Seasonally, FPP per unit area peaked in summer (July–August: 30–50 g/m^2^) and declined in spring (May–June: 0–30 g/m^2^). Spatially, the highest values occurred near riverbank and tributary estuaries, whereas central waters remained low. The FCCI revealed significant heterogeneity; the northwestern regions experienced high food demand pressure (FCCI > 0.5), while the southeastern areas were underutilised (FCCI < 0.3). As the lake‐wide FCCI never exceeded 0.6, the current level of primary productivity can support further strategic restocking, provided that releases are redirected to the southeast to relieve pressure on the northwest. This study demonstrates how remote sensing can be used to balance fish conservation and production goals in sensitive plateau ecosystems.

## Graphical abstract

This study evaluates fish potential production (FPP) and fish carrying capacity index (FCCI) in Qinghai Lake using satellite‐based primary production estimates. Results show increasing FPP trends and spatially heterogeneous FCCI, indicating potential for sustainable fisheries management and guiding restocking efforts in this high‐altitude lake ecosystem.

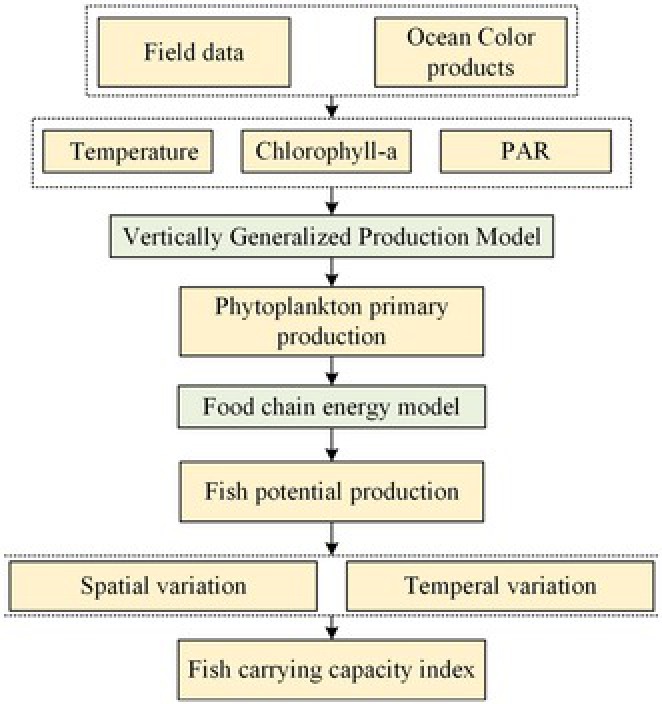

## 1 Introduction

Global fisheries face unprecedented challenges in balancing sustainable yields with ecosystem conservation, particularly in alpine lakes where climate change is shortening ice cover and amplifying interannual variability in phytoplankton primary production (PP) (Silsbe et al. 2025). Fish potential production (FPP)—the biomass that PP can sustain without degrading future ecosystem services—has become a central metric for quantifying these energy constraints (Pauly and Christensen 1995). Conventional FPP estimates rely on sparse field measurements of PP and assumed trophic transfer efficiencies (Rosenberg et al. 2014), approaches that are logistically prohibitive across the vast, wind‐swept surfaces of high‐altitude lakes(Rosenberg et al. 2014; Song et al. 2012; Wang et al. 1994). Conventional PP measurement methods such as the Winkler technique, while accurate, require costly and time‐consuming field campaigns that are logistically impractical for dynamic, large‐scale and long‐term monitoring (Johnson and Witney 1939). This limitation is particularly acute in remote and extreme environments (Fogarty et al. 2016).

Qinghai Lake, located on the Tibetan Plateau at an altitude of 3260 m, is the largest inland saltwater lake in Asia. This high‐altitude lake is characterised by its unique ecological features, including a semi‐arid climate and high salinity and alkalinity, making it one of the most fragile ecosystems in the world (Jin et al. 2009). The lake contains the world's only self‐sustaining population of naked carp ( *Gymnocypris przewalskii* ), a keystone species that supports the food web of the plateau and is now a second‐class nationally protected species in China. The population of naked carp dominates Qinghai Lake, accounting for over 95% of its total fish biomass (Zhou et al. 2016; Zhou et al. 2021). However, severe overfishing in the late 20th century caused the population to collapse from 200,000 t (1960s) to 2592 t (2002), disrupting the ecological equilibrium (Jianquan 2008). Since 2002, fishing bans and artificial restocking have driven a partial recovery (~100,000 t in 2024) (Shi et al. 2016). However, managers still lack quantitative tools to determine how much PP is available to sustain further fish population growth, and where in the 4500 km^2^ lake restocking efforts should be concentrated. Sustainable management of this recovering population requires precise quantification of energy thresholds through FPP, integrating PP pathways and population dynamics to optimise restocking strategies and resource allocation in this climate‐sensitive system.

Understanding the spatiotemporal variation of FPP in Qinghai Lake is essential for deciphering the population dynamics of the naked carp and ecosystem resilience, but such studies face significant methodological and observational challenges (Deng et al. 2017; Guan et al. 2005; Li et al. 2020). Existing FPP assessments in Qinghai Lake have relied on short‐term field surveys (2006–2010) (Jia et al. 2021; Yao et al. 2011), which are logistically unfeasible for large‐scale, long‐term monitoring due to the vast size and extreme climate of the lake. Consequently, these studies lack multidecadal FPP sequences necessary to detect climate‐driven cycles. Although satellite remote sensing has been applied to study environmental parameters such as water level, ice duration and temperature in Qinghai Lake (Cai et al. 2017; Shi et al. 2020; Zhang 2011), its integration with PP‐to‐FPP modelling remains unexplored for alpine lake systems. Furthermore, widely used satellite‐based PP models like the Vertically Generalised Production Model (VGPM) lack specific adaptations for high‐altitude lakes (Behrenfeld and Falkowski 1997). Critically, no previous study has quantified how FPP variability influences fish carrying capacity or adaptive fisheries strategies—a fundamental knowledge gap impeding sustainable governance in this fragile ecosystem.

This study addresses these gaps by developing a novel framework that integrates satellite‐derived PP (via MODIS chlorophyll *a*) with food chain energy transfer models, enabling high‐resolution (1 km^2^) and long‐term (2002–2024) monitoring of FPP in Qinghai Lake. A key innovation is the development of the Fish Carrying Capacity Index (FCCI), the first index tailored to alpine lake ecosystems that explicitly links variation in FPP to fish population (FP) sustainability and stocking strategies. Unlike conventional PP–biomass ratios, the FCCI provides a spatially explicit, time‐varying threshold metric that directly informs management actions such as zonal restocking and harvest control. By combining satellite‐based VGPM data with in situ surveys, we achieve two breakthroughs: (1) overcoming the limitations of laboratory‐based or low‐resolution measurements by estimating trophic transfer efficiency in a high‐altitude system; (2) establishing quantitative decision‐making tools for spatial hotspot identification and fishing pressure thresholds to guide zone‐specific management.

Our approach is conceptually transferable to other endemic, cold‐water systems such as Lake Titicaca, Andean plateau lakes and African rift lakes, where single‐species dominance and remote sensing accessibility meet similar monitoring challenges (González et al. 2025; Loayza et al. 2023; Plessl et al. 2017). Moreover, the FCCI framework explicitly incorporates climate sensitivity by tracking how temperature shifts, ice cover duration and nutrient fluctuations—key drivers of PP in alpine lakes—cascade through the food web to affect fishery potential (Xu et al. 2024). This work establishes a new paradigm for alpine lake monitoring and provides actionable methods for balancing conservation and sustainable fisheries management in Qinghai Lake, offering a replicable model for fragile ecosystems worldwide under accelerating climate change.

## 2 Data and Methods

### 2.1 Study Area

Qinghai Lake, situated on the Tibetan Plateau at an altitude of 3260 m. The lake covers an area of approximately 4500 km^2^ and a depth of about 20 m. The lake area has a semi‐arid continental plateau climate with abundant light, cold winters, cool summers and low rainfall, being influenced by monsoons and westerlies, making the environment sensitive to climate change (Jin et al. 2009). There are five permanent tributaries that provide about 80% of the total water inflow to Qinghai Lake. The lake water is stratified in summer (hypolimnion < 6°C, epilimnion 12°C–15°C) and freezes from November to April (Wang et al. 2016). The naked carp of Qinghai Lake live only in the lake water, even though the water is highly saline and alkaline. The fish migrate to the freshwater tributaries to spawn from May to September. Naked carp has gradually become a native fish unique to the lake, forming the dominant population, accounting for more than 95% of the total fish resources in Qinghai Lake (Zhou et al. 2016).

### 2.2 Data Sources

#### 2.2.1 Field Data

**FIGURE 1:**
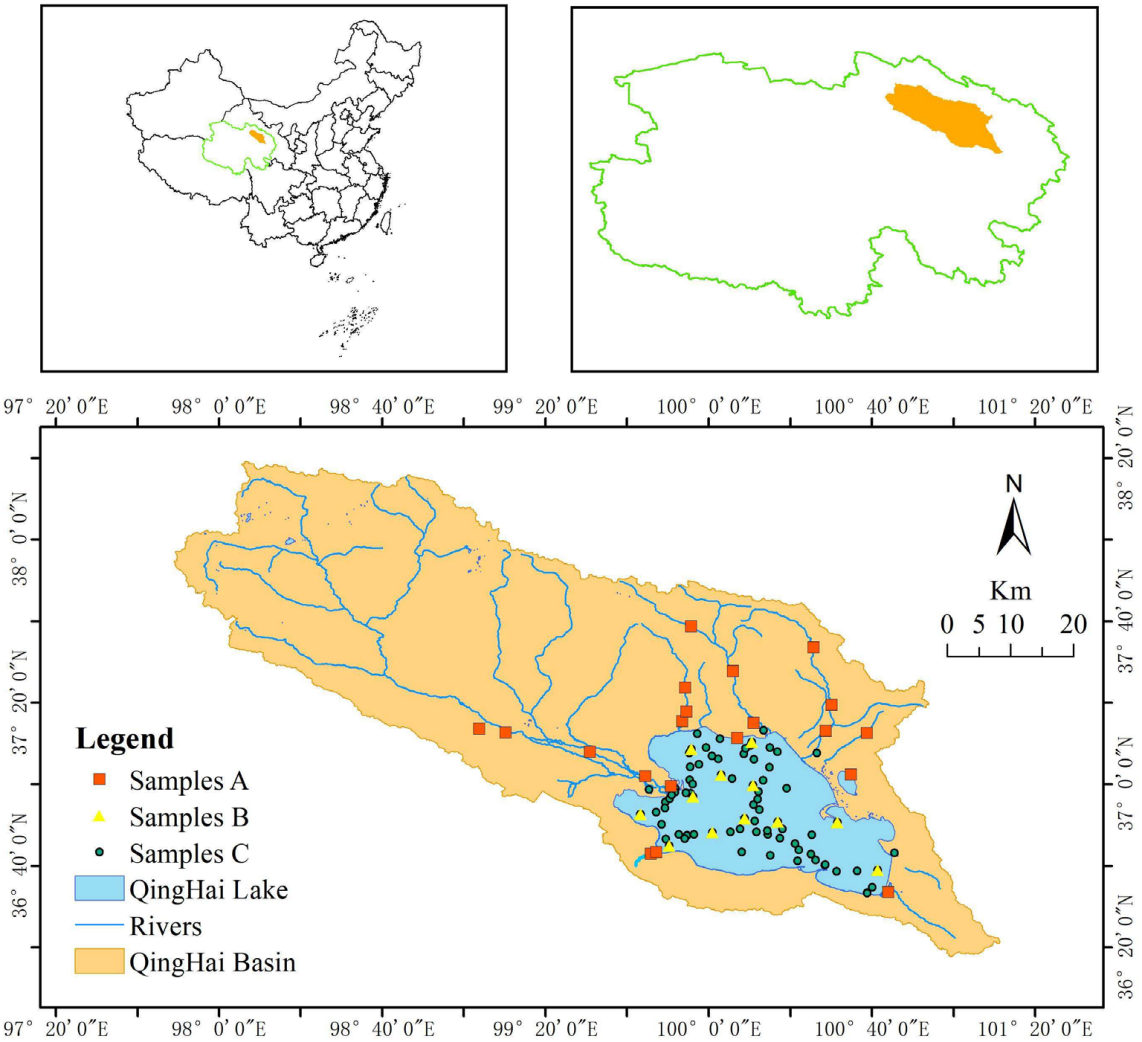
Location of Qinghai Lake and Sample Sites. Samples A are the biological samples collected in the tributaries of Qinghai Lake, the naked carp spawning grounds; Samples B are the biological samples collected in Qinghai Lake; Samples C are the environmental samples collected in Qinghai Lake during 2018–2024.

Between 2018 and 2024, twelve survey cruises were conducted to monitor the water temperature (T), chlorophyll *a* (Chl‐a), phytoplankton density and fish population in Qinghai Lake from May to October (Figure 1, Table S1). Phytoplankton samples were collected at three depths (0.5 m below the surface water, mid‐depth and 0.5 m above the lake bottom) using a Plexiglas sampler and fixed with Lugol's solution for laboratory analysis (Samples A and B in Figure 1). PP was estimated using the Winkler method (Macedo et al. 1998; Carignan and Blais 2011). Environmental samples were also collected throughout the lake (Samples C in Figure 1). T and Chl‐a were measured at 0.5 m under the lake surface using a portable multiparameter water quality analyser (EXO3, YSI).

Annual FP was quantified using a dual‐method approach (Chen et al. 2009; Wang et al. 2013). (1) Acoustic surveys: lake‐wide hydroacoustic surveys were conducted in August (the season of peak biomass) using a BioSonics DT‐X scientific echo sounder (EY60, SIMARAD) at 210 kHz. The survey route covered the entire lake (4500 km^2^) (Figure 1), with GPS‐tracked navigation ensuring spatial consistency. Fish density (individuals/m^3^) was derived from echo‐integration using BioSonics Visual Analyser software (v4.3) and was calibrated against in situ gill‐net catches. (2) Net sampling and calibration: 200‐m multi‐mesh gill nets (mesh sizes: 10–100 mm) were simultaneously deployed at 12 representative sites (Figure 1 sample B). The captured naked carp were measured (length and weight) to establish species‐specific target strength (TS) relationships:

(1) $$\mathrm{TS}=19.1\times \log_{10}\left(L\right)-0.9\times \log_{10}\left(f\right)-62$$

where TS represents the target strength value (dB); *L* represents the target fish body length (cm); *f* represents the frequency of the echo sounder transducer (kHz), which was set to 210 kHz in this study.

Lake‐wide FP (tonnes) was calculated as:

(2) $$\begin{array}{c} \mathrm{FP}=\text{Mean density}\;\left(\mathrm{ind}./m^{3}\right)\times \text{mean weight}\;\left(g\right)\\ \;\times \text{lake volume}\left(\;,m^{3}\right)\times 10^{-6} \end{array}$$

Validation against mark‐recapture data (2018–2024, *n* = 12 survey campaigns) showed a mean error of ±7.2% (see Data S2).

#### 2.2.2 Satellite and Meteorological Data

Satellite‐based estimates of T and Chl‐a concentration were derived from MODIS products (Table S2). The T estimates produced by the proto‐algorithm were labelled version 1. This was a Level 2 product with EOS DIS product number 2527, MODIS product number 28, labelled Sea Surface Temperature. Chl‐a (MOD21, Chlor_a_3) and instantaneous PAR (MOD22) were used to estimate the PP value of Qinghai Lake from May to October in 2002–2024. Daily Level‐2 data from MODIS‐Aqua and MODIS‐Terra corresponding to the survey dates were downloaded from the NASA Ocean Color website (http://oceancolor.gsfc.nasa.gov). Full resolution (1 km) local area coverage images of Qinghai Lake were processed using Python, and the following products were obtained: Chl‐a using the OC3Mv6 algorithm; Kd_(PAR)_ using Lee's model for Kd_(490)_ to estimate the euphotic depth (Lee et al. 2013), and the sea surface temperature daytime product for estimating T (11‐μm band) (Table S3). A total of 33,580 satellite datasets spanning 23 years from 2002 to 2024 were used to estimate PP in Qinghai Lake. The valid data were defined as pixels with Chl‐a retrieval flags = 0 (nominal), Straylight risk < 5% (the MODIS L2 product) and Ice cover = 0 (confirmed by the MOD10A1 product) after applying the above quality filters. To mitigate temporal sampling bias, monthly PP estimates were computed only for pixels with ≥ 5 valid days per month, and values were gap‐filled using linear interpolation for periods with missing data (< 10% of months). To maintain spatial consistency across the time series and mitigate edge effects, a unified lake mask was created based on the minimum water extent (2002–2024) and applied to all data. This conservative approach excludes intermittently wet pixels (~3.5% of the total), ensuring that FPP trends are not confounded by fluctuating lake boundaries.

### 2.3 Methodology

Satellite‐based estimates of PP using the VGPM were combined with more complete food chain approaches to estimate the FPP of naked carp in Qinghai Lake. VGPM was chosen following a rigorous evaluation against the Carbon‐based Productivity Model (CbPM) and the Eppley‐VGPM Hybrid (Li et al. 2020; Lv et al. 2025). The CbPM's reliance on particulate backscattering was considered unreliable due to the presence of glacial flour in the tributary estuaries of Qinghai Lake that contaminated the signal. MODIS imagery from 2018 to 2020 revealed that the CbPM yielded an RMSE that was 32% higher than the value from VGPM (23.1 mg C m^−2^ day^−1^ versus 12.7 mg C m^−2^ day^−1^), primarily due to flawed retrieval near turbid inlets such as the mouth of the Buha River. Meanwhile, the Eppley‐VGPM Hybrid overestimates PP by 40%–60% in oligotrophic waters because its temperature‐driven growth assumption disregarded nutrient limitation. With phosphate levels below 0.05 mg L^−1^, the phytoplankton in Qinghai Lake remain constrained by the scarcity of phosphorus, even under optimal temperatures. VGPM outperformed both models by explicitly parameterising $P_{\mathrm{ept}}^{B}$ as a joint function of temperature and chlorophyll *a*. This approach captures the lake's diurnal thermal swings from 0°C to 25°C without disregarding the nutrient constraint.

**FIGURE 2:**
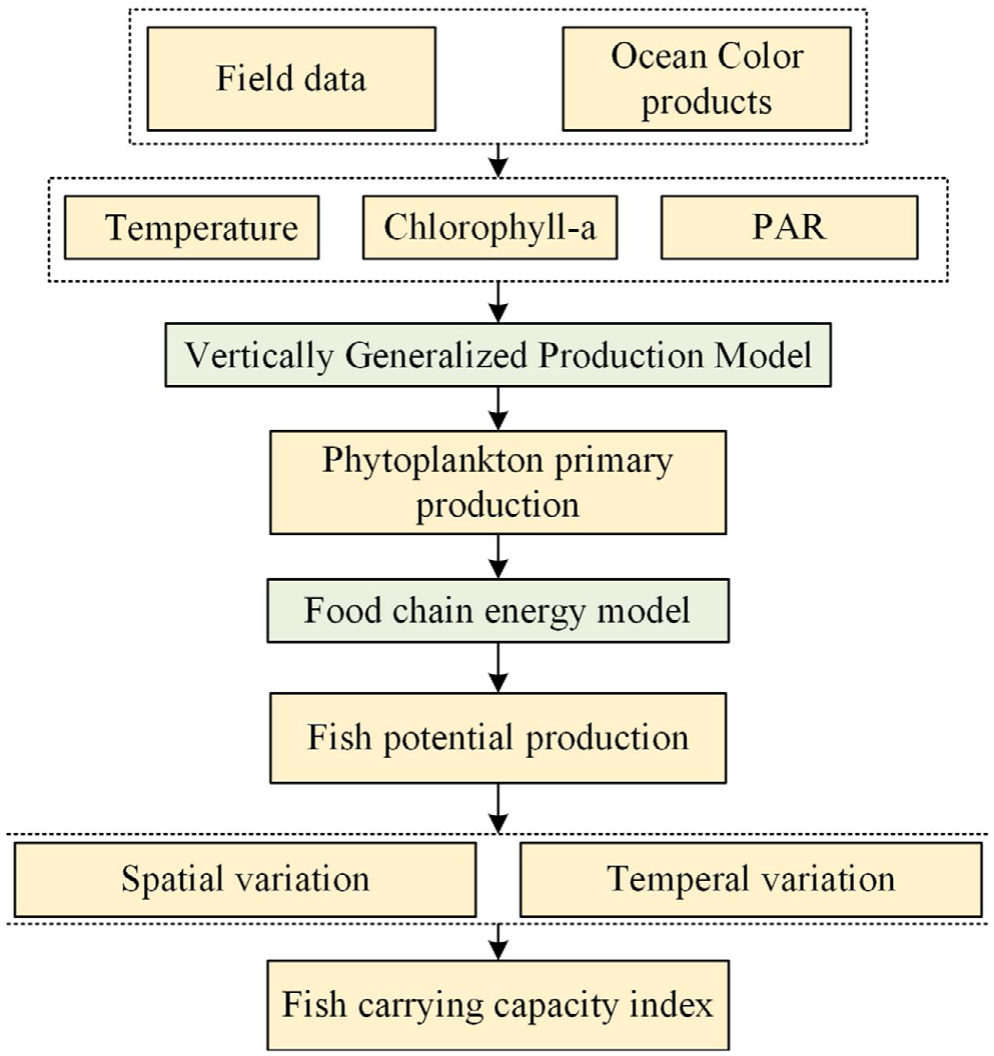
Framework for calculating potential fish production and the fish carrying capacity index.

The FPP was combined with in situ catch and fish echo sounder survey data to calculate FCCI. The spatial and temporal analysis of FPP and FCCI could be used to guide restocking programmes for ecosystem conservation. The framework employed to calculate FPP and FCCI is shown in Figure 2.

#### 2.3.1 Calculation of Potential Fish Production

The FPP was obtained by energy transfer modelling from primary producers through the food chain and the PP needed to sustain the fisheries. This method, based on Pauly and Christensen's approach, estimates the FPP required by fisheries using the trophic level of caught species, energy transfer efficiency and PP (Pauly and Christensen 1995). Net PP in the surface water layer was calculated from the satellite data using the VGPM model. Details of the VGPM equation and parameters are provided in the Supporting Information and Table S3 (Behrenfeld and Falkowski 1997; Gaoping and Liangqiang 2004).

FPP is based on the concept that exploited species need a minimum supply of organic energy to replenish themselves. This energy, originating from solar energy captured by autotrophs such as phytoplankton, moves through the food chain to the secondary producers, planktivorous consumers and predators (Leach et al. 1987). At each trophic level, only a fraction of the energy is used for growth; this is known as transfer efficiency and depends on factors including search effort, metabolism and assimilation efficiency (Collie et al. 2009). Thus, calculating FPP requires knowledge of a species' trophic levels and assumptions concerning transfer efficiency (Baumann 1995). This study implemented a three‐tier FPP classification system that distinguishes spatial resolution and aggregation scales (Table 1). The spatial resolution of net FPP enables the detection of local hotspots, and lake‐wide aggregation (tonnes) supports fisheries management.

**TABLE 1:** Fish Potential Production (FPP) classification and definitions used in the study.

FPP type	Definition	Units	Calculation
Net FPP	Per‐grid‐cell FPP	g/m^2^	$\mathrm{Net}\;\mathrm{FPP}=\frac{\mathrm{Net}\;\mathrm{PP}\times a^{n}\times \left(P/B\right)}{E}$
Gross Monthly FPP	Lake‐wide Net FPP sum for a single month	Tonnes	Gross Monthly FPP = $\left(\frac{1}{n}\sum \mathrm{Net}\;\mathrm{FPP}\right)\times A_{\text{lake}}\times 10^{-6}$
Gross Annual FPP	Cumulative May–October Gross Monthly FPP sum	Tonnes	Gross Annual FPP = $\sum_{\mathrm{May}-\mathrm{Oct}}\text{Gross Monthly}\;\mathrm{FPP}$

Net FPP is the per‐grid‐cell fish potential production (g/m^2^); Net PP is the per‐grid‐cell phytoplankton primary productivity (mg C/m^2^); a is the trophic transfer, which is 10% in the Qinghai Lake, which was adopted and specifically validated for Qinghai Lake, who reported minimal seasonal variation (±2%) due to the lake's simplified food web and the stenophagous diet of naked carp (Downing et al. 1990); n is the trophic level. Naked carp occupies a fixed trophic level (*n* = 2) as obligate zooplanktivores, with gut content analyses confirming that zooplankton comprises 95% of their diet (Zhou et al. 2021). This dietary specialisation persists across seasons because Qinghai Lake's oligotrophic conditions limit the availability of alternative prey; phytoplankton and detritus constitute < 5% of total intake (Luo et al. 2019). Despite spawning migration to tributaries (May–September), stomach sampling has confirmed consistent zooplankton dominance in summer (87%–92%) and winter (94%–97%) (Tian et al. 2019; Xie et al. 2023; Yao et al. 2011; Zhou et al. 2016). P/B is the ratio of PP to its biomass (ranging from 30 to 50); this is typically equal to 30 in Qinghai Lake (Yao et al. 2011). E is the efficiency of energy conversion; 1 g fresh weight of naked carp equals 2.5 kJ (1 kJ = 1 mg C/m^2^) (Zhou et al. 2021), and therefore, E equals 2.5 for naked carp. A_lake_ = 4500 × 10^6^ m^2^ in the Qinghai Lake area. The Gross Monthly FPP (tonnes) is the lake‐wide vet FPP sum for a single month. The Gross Annual FPP (tonnes) is the cumulative May–October Gross Monthly FPP. The units transition from primary productivity (mg C m^−2^ day^−1^) to fish potential production (g m^−2^ month^−1^) is showed in Table S5.

#### 2.3.2 Calculation of the Fish Carrying Capacity Index

In this study, we developed an FCCI to provide threshold information for fisheries management. We defined FCCI as the maximum population of fish that can be supported indefinitely in a given water body under ideal natural conditions without artificial feeding or fertilisation. The concept is closely related to the sustainability of fishing activities and can be used to determine rational stocking, harvesting and utilisation of natural bait resources. Calculation of the FCCI was performed by comparing satellite‐derived spatial estimates of FPP with the annual increment of FP estimated by in situ catch using fish echo sounder surveys in August:

(3) $$\text{FCCI}=\frac{\mathrm{FP}}{\text{Gross Annual}\;\mathrm{FPP}}$$

where FP denotes the annual, protocol‐standardised discrete measurements (Data S2). These were derived from field surveys carried out every August (2002–2024) using hydroacoustic transects and gill‐net sampling. This time period was chosen to capture the peak biomass of naked carp during their spawning migration.

The FCCI could be used to guide the management of the fishery carrying out the naked carp restocking measures, where an index value of is less than or equals to 1 would indicate that the available PP exceeds the energy demand of the fish population, suggesting underutilised resources and potential for sustainable population growth (i.e., that by restocking the fish population could be sustained in the absence of other species preying on the fishery), and the closed fishing and restocking measures should continue. An index value is greater than 1 would indicate overexploitation of the fishery resource, where fish biomass exceeds the supporting capacity of the ecosystem, so that the fishery biomass would be expected to decrease over time. This could be observed by a decline in PP due to a decrease in the mean trophic level. Failure to observe such a decrease would suggest that the fishery is being supported by a higher rate of PP than is observed. The FCCI could be used to guide spatial management rules for restocking fish in Qinghai Lake, and it has been suggested that restocking fish should be released in those areas where the value of FCCI is less than 1. It is important to note that accurate estimates of PP and detected FP are essential for an accurate assessment of FCCI.

#### 2.3.3 Model Evaluation and Verification

To evaluate the satellite products and the results of the VGPM model, the median of each product was extracted in a 1 × 1 km pixel box centred on the location of the sampled sites with a ±12 h time difference between the satellite passes to compare with the field survey results for Chl‐a, T and PP. The ±12 h window accounts for MODIS fixed overpass limitations: the Terra satellite crosses Qinghai Lake at 10:30 AM local time, Aqua at 1:30 PM local time (exactly two overpasses/day) (Xu et al. 2017). Field sampling (2018–2024) spanned 6:00 AM–6:00 PM to capture diurnal variability, but the schedule could not align with the MODIS overpasses for all sites. Ecologically, this window is justified because phytoplankton dynamics in high‐altitude lakes exhibit < 5% diurnal variation in chlorophyll *a* (Li et al. 2016). PP integrates light exposure over 4–6 h, minimising sub‐daily variation (Zhao et al. 2003).

The quality of the satellite images retrieved was assessed using the standard masks and flags, with the criterion that the standard deviation should be less than 20% of the mean to be considered a valid match.

To assess the overall performance of the model and algorithm, the average relative error (ARE) was calculated as a measure of accuracy; the correlation coefficient (CC) was used as a measure of the association between the modelled and field data; the root mean square error (RMSE) was used as a measure of the average error size, and the accuracy of estimation was used as a measure of precision. These parameters are defined as:

(4) $$\mathrm{ARE}=\frac{1}{n}\sum \left(\frac{X_{m}-X_{s}}{X_{s}}\right)\times 100$$

(5) $$\mathrm{CC}=\frac{\mathrm{Cov}\left(X_{m},X_{s}\right)}{\sqrt{\mathrm{Var}\left[X_{m}\right]\mathrm{Var}\left[X_{s}\right]}}$$

(6) $$\text{RMSE}=\sqrt{\frac{1}{n}\sum {\left(X_{m}-X_{s}\right)}^{2}}$$

(7) $$\mathrm{EA}=\left(1-\frac{\text{RMSE}}{\bar{X_{s}}}\right)\times 100\%$$

where *X_m_* and *X_s_* are the modelled and surveyed variables analysed, respectively, and *n* is the number of samples.

## 3 Results

### 3.1 Validation of Satellite Products and VGPM Result

**FIGURE 3:**
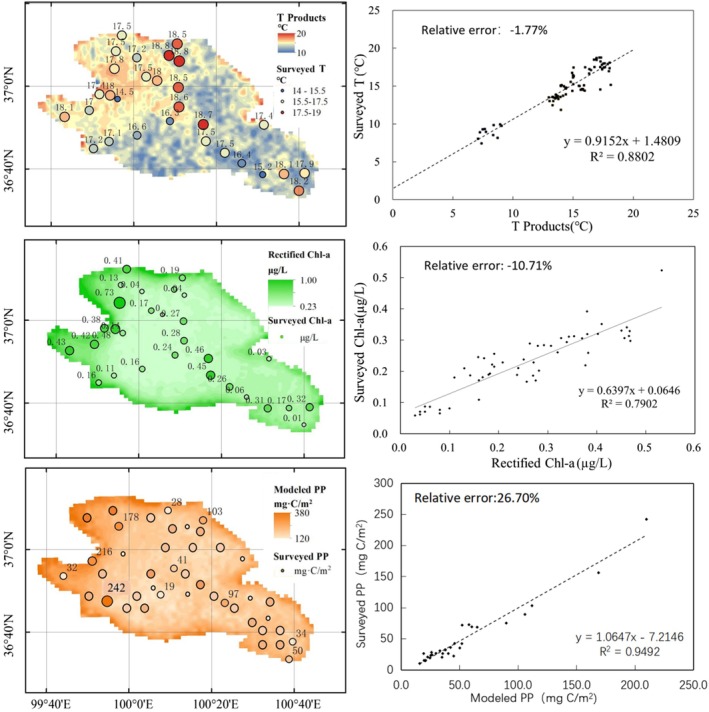
Comparison of the modelled and measured parameters in Qinghai Lake. (a) surface temperature from Ocean Colo; (b) rectified chlorophyll a; (c) VGPM‐modelled phytoplankton primary production.

The estimation of FPP relies on the estimates of PP calculated using the VGPM model. The parameters of the VGPM model and the PP estimates were validated against the survey data. From 2018 to 2024, 4230 grid cells from satellite products and VGPM results were compared with 316 surveyed data points for T, Chl‐a and PP. Scatter plots and error analysis are shown in Figure 3 and Table S4, respectively. To ensure independence between calibration and validation, Chl‐a and PP regression was performed using samples from 2018 to 2019, whereas validation was based solely on measurements from 2020 to 2024 (*n* = 316), with no temporal overlap between the two datasets.

A type II linear regression model was used to compare the satellite‐derived and VGPM‐modelled values with field data. The comparison showed a strong correlation, with Pearson correlation coefficients (CC) ranging from 0.79 to 0.97. For temperature, the CC was 0.88, and the EA was 92.64% (Figure 3a). However, the ARE for Chl‐a was high, indicating overestimation. This was corrected by a regression method using 57 measured Chl‐a samples for construction of the regression equation and 20 measured samples for calibration. The corrected Chl‐a values showed an ARE of 10.71% and an EA of 72.29% (Figure 3b). Model calibration used 41 in situ PP measurements collected during 2018–2024 from the Buha River estuaries and central deep zones to littoral areas in Qinghai Lake. CC = 0.95, EA = 75.97%, RMSE = 11.61 mg C m^−2^d^−1^ and ARE = 26.70%. The spatial fidelity of modelled and measured PP values aligned closely across oligotrophic central waters and eutrophic estuarine regimes (Figure 3c). The spatial distribution of T and the corrected Chl‐a values showed similar trends, with higher values near the shoreline. The spatial distributions of measured and modelled PP also showed good agreement, indicating that the VGPM model accurately predicted PP in Qinghai Lake.

### 3.2 Seasonal and Spatial Variation in Fish Potential Production

**FIGURE 4:**
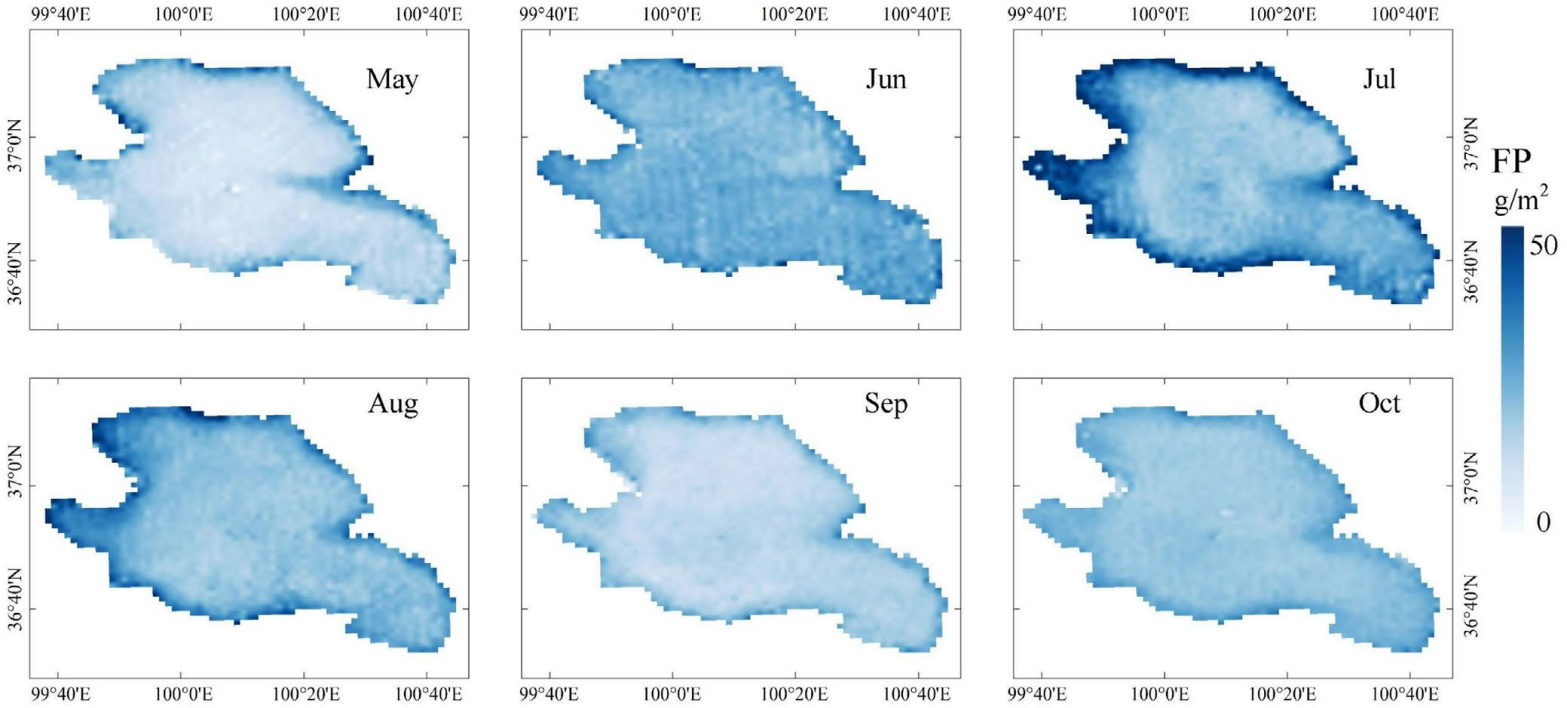
Spatial distribution of potential production in the non‐freezing period of Qinghai Lake (MODIS‐Aqua results in 2020).

The spatial distributions of the FPP in Qinghai Lake were calculated from the VGPM model, and the food chain energy transfer equation was used to analyse the seasonal variation. Taking the 2020 results as an example, the spatial distribution of FPP was generally light in the centre of the lake and greater near the shore and at the mouths of Qinghai Lake's tributaries during the non‐freezing period (May–October) (Figure 4). The FPP increased from May to July and then decreased from July to October; the highest values generally appeared in summer, approximately 30–50 g/m^2^ per grid unit (July and August) with high spatial heterogeneity (the red areas in Figure 4), while the lowest values generally appeared in spring (May–June), at about 0–30 g/m^2^ per grid unit with greater uniformity (the blue areas in Figure 4). The FPP in other years had similar seasonal and spatial trends.

**FIGURE 5:**
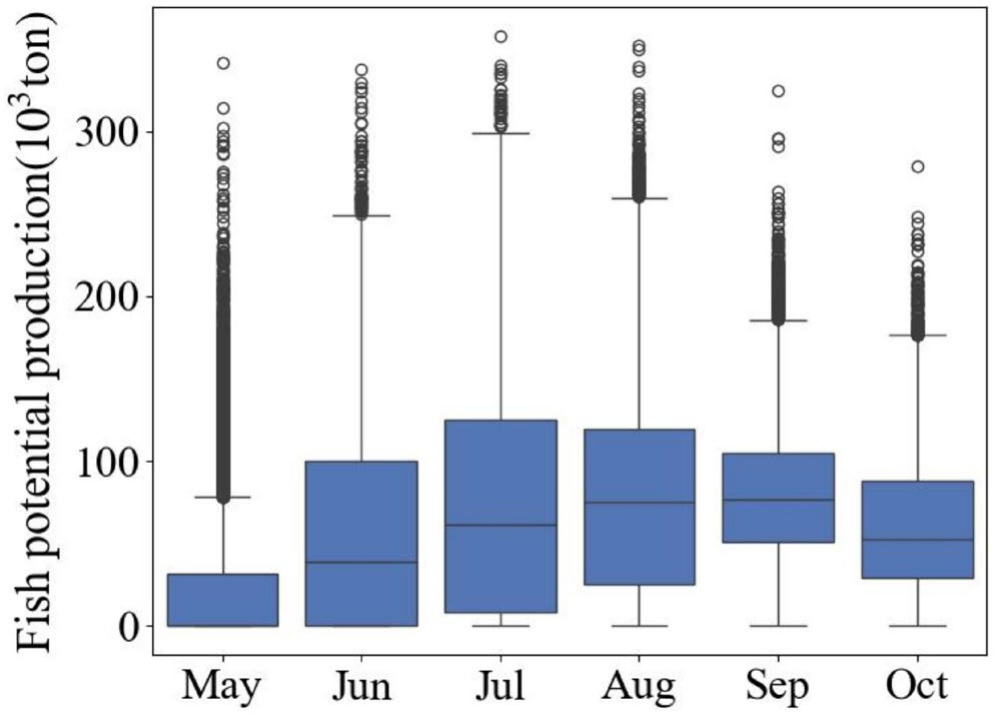
Box plots of the monthly average fish potential production from 2002 to 2024 in the unfrozen period of Qinghai Lake (results from MODIS‐Aqua).

Box plots of the median average monthly FPP from 2002 to 2024 showed an increasing trend from May to September (Figure 5), with September having the highest median production of around 80,000 t; there was significant variability, as indicated by long whiskers and several outliers in the plot. August maintained a similar production level, but with slightly reduced variability. October exhibited a further drop in production to a median of 50,000 t. The box plots provide a clear view of the median, interquartile range and outliers, highlighting the changes in average monthly FPP stability and variability, indicating the most consistent and highest production during the summer, with variability decreasing as the season progresses.

### 3.3 Annual and Spatial Variation in Fish Potential Production

**FIGURE 6:**
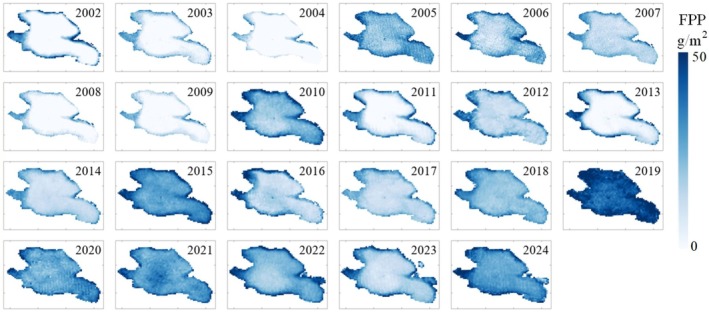
Spatial distribution of fish potential production from 2002 to 2024 in Qinghai Lake in August.

The FPP of Qinghai Lake was estimated annually from 2002 to 2024 during the non‐freezing period, with a focus on August (Figure 6). Naked carp migration to tributaries (May–September) concentrates the biomass in August, minimising spatial heterogeneity and maximising detectability (Zhou et al. 2021). August is a critical month for naked carp, making it representative for the estimation of FPP. The values of FPP were significantly higher near the shore, especially in the northwestern region near the Buha and Quanji River estuaries. From 2002 to 2004, FPP values were generally low (< 5 g/m^2^). FPP increased to 5–30 g/m^2^ in 2005–2007, declined to below 5 g/m^2^ in 2008–2009, and significantly rose in 2010, especially near the mouths of the Buha and Quanji Rivers. The FPP values decreased to below 15 g/m^2^ from 2011 to 2014, slightly increased to 25–40 g/m^2^ in 2015, fell to 10–25 g/m^2^ in 2016–2018, peaked at 40–50 g/m^2^ in 2019, dropped to 20–40 g/m^2^ in 2023 and increased again in 2024. Annual FPP values varied from 0 to 50 g/m^2^, showing a periodic pattern and a peak in 2019.

**FIGURE 7:**
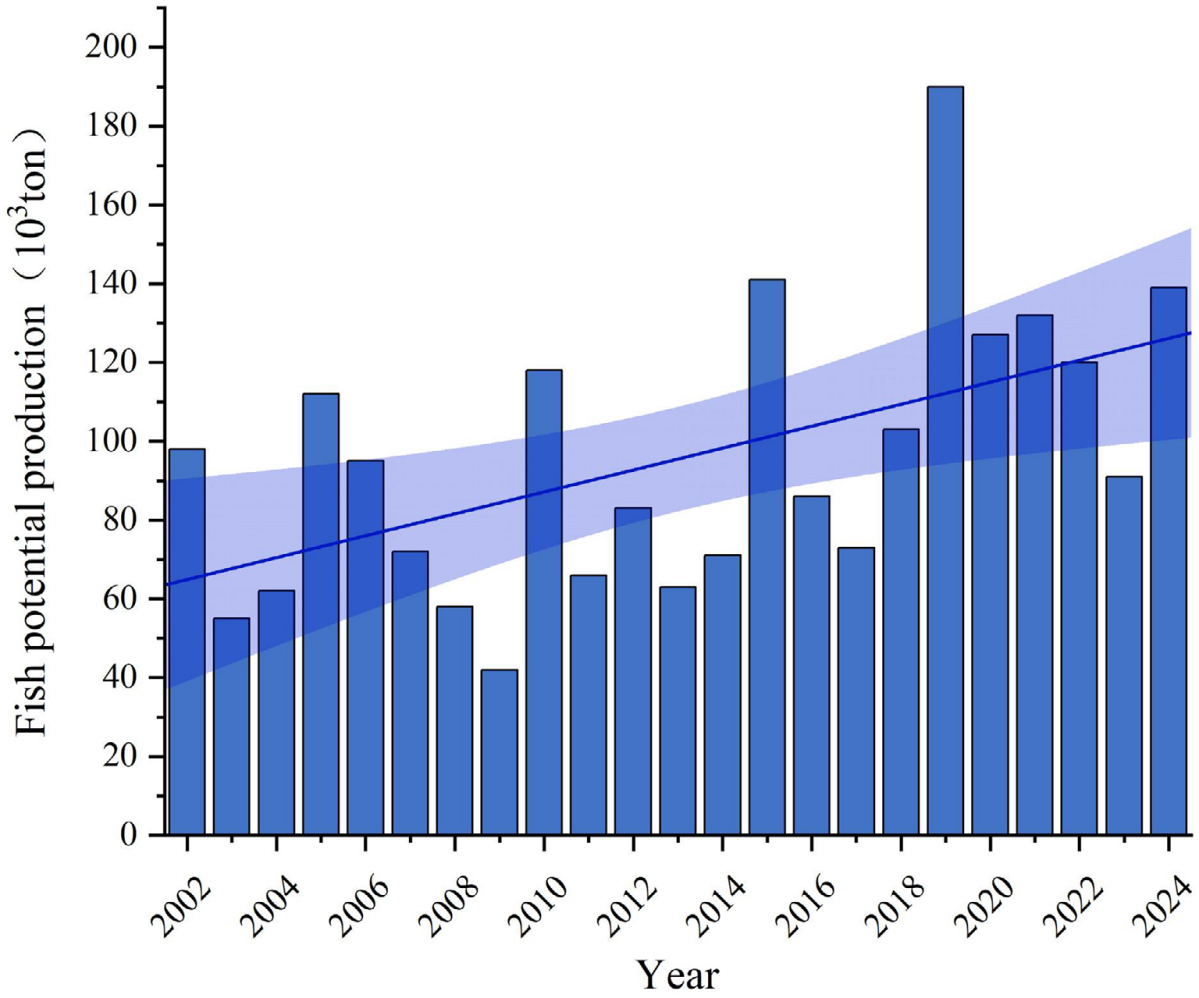
Gross fish potential production of Qinghai Lake from 2002 to 2024 in August of each year.

The gross FPP in Qinghai Lake fluctuated between 42,000 and 176,000 t from 2002 to 2024 (Figure 7). There was an upward trend until 2010, followed by a decrease in 2011, a recovery period lasting until 2016 and a dramatic increase in 2019. After 2019, there was a decline, but the overall value remained high. From 2020 to 2024, FPP initially increased and then decreased, peaking in 2019.

### 3.4 Fish Carrying Capacity Index for Naked Carps in Fisheries Management

**FIGURE 8:**
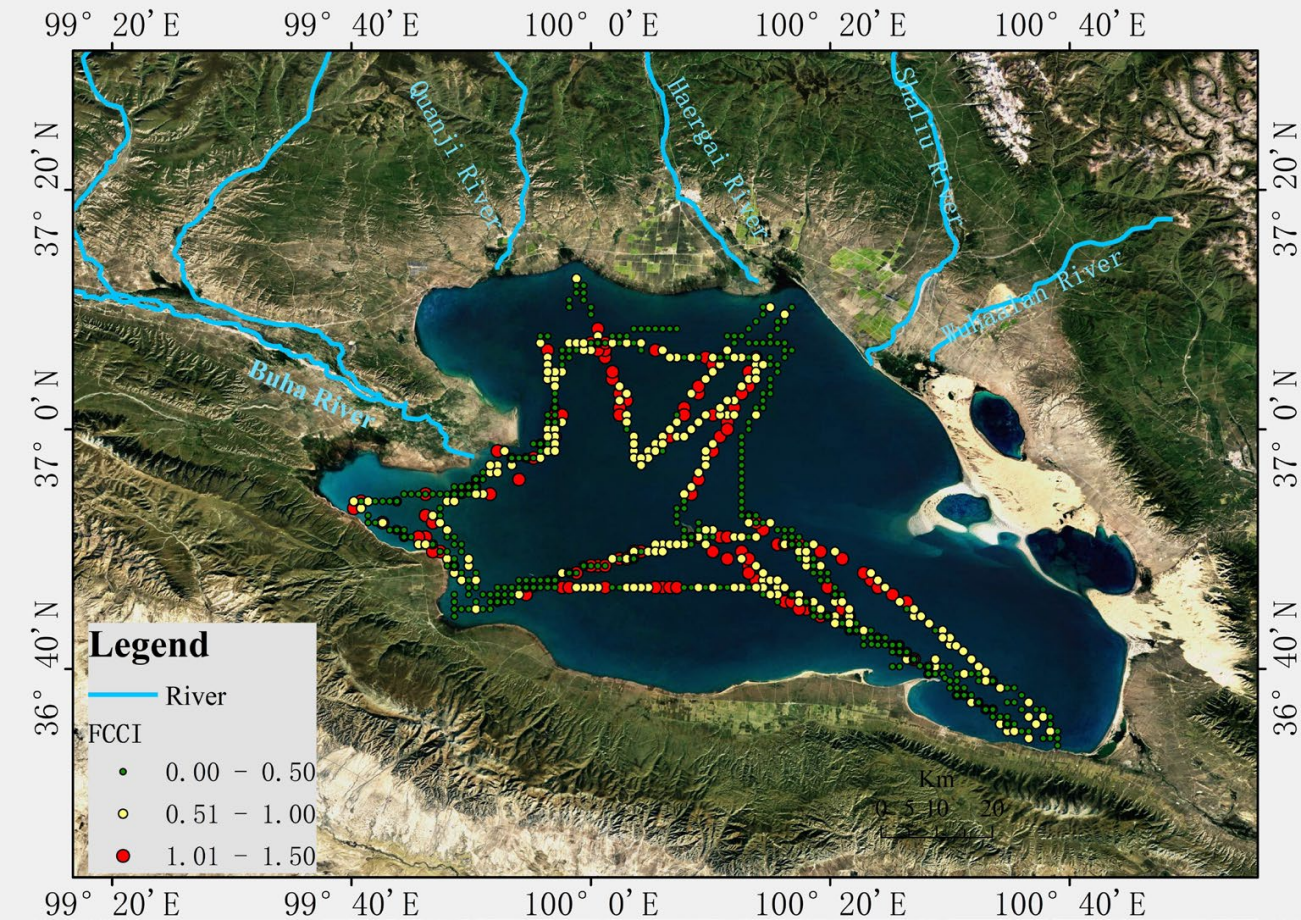
Distribution of the fish carrying capacity index in different years.

**FIGURE 9:**
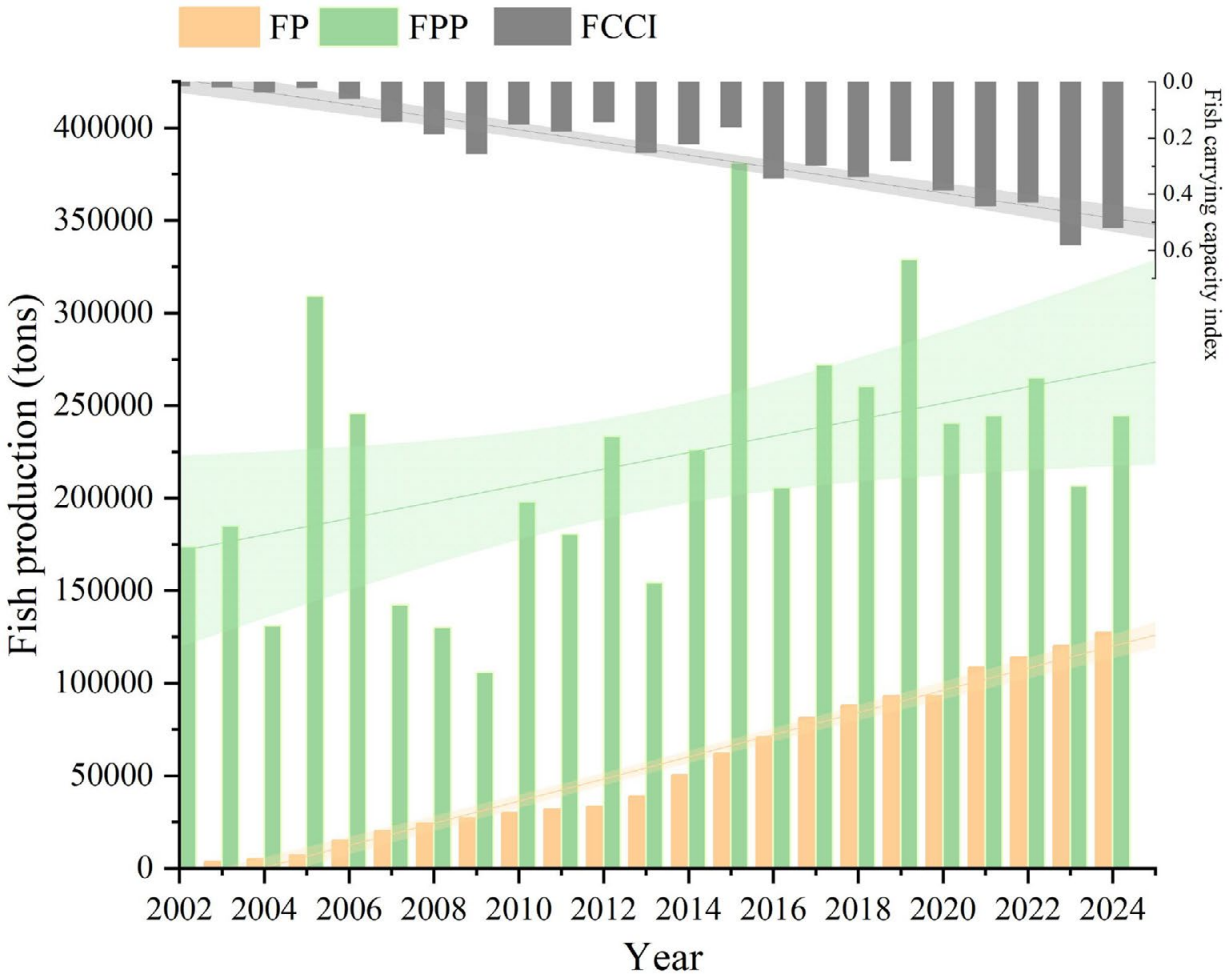
Total annual fish production, fish potential production and fish carrying capacity index of Qinghai Lake.

The FCCI results showed that most values in Qinghai Lake were below 1, suggesting that PP, as a natural food source, is adequate for the growth and maintenance of the naked carp population. While FCCI < 1.0 indicates that PP exceeds fish energy demand in this simplified food web, other factors such as oxygen saturation, habitat availability and interspecific competition are minimal in Qinghai Lake due to the dominance of naked carp and the lake's oligotrophic, well‐oxygenated conditions. The spatial distribution of FCCI in Qinghai Lake exhibited significant heterogeneity (Figure 8). Notably, higher FCCI values were observed in the northwestern regions and along the shores and estuaries of the lake. This spatial analysis points to a relatively higher food resource pressure for the naked carp in the northwestern part of the lake near the restocking streams and migration routes of the species. The elevated FCCI in these areas, despite the high PP, suggests that the restocking and migration activities of naked carp significantly impact the northwestern region's fish community composition. Given this, it is recommended that future restocking activities be implemented in the southeastern areas of Qinghai Lake, where the FCCI values are lower, potentially balancing the resource pressure and supporting a more sustainable fishery management strategy. The annual FCCI measures the annual FP against FPP in Qinghai Lake (Figure 9). FP increased from 2592 t in 2002 to 127,500 t in 2024, while FPP peaked at 400,000 t in 2015, stabilising at 250,000 t by 2024. FCCI has increased, but it remains below the 0.6 benchmark, indicating substantial potential for the growth of the fish population through restocking. Note that 0.6 is not an ecological threshold; it is merely the level above which global fisheries have frequently become overexploited (Knight and Jiang 2009). The FCCI threshold of 1.0 represents the theoretical maximum sustainable yield (Pauly and Christensen 1995; Rosenberg et al. 2014).

### 3.5 Sensitivity Analysis of FPP and FCCI to Parameter Variation

To evaluate the impact of parameter uncertainty on the estimates of FPP and FCCI, we conducted a Monte Carlo sensitivity analysis (1000 iterations) with ±20% variation in four key parameters: Trophic transfer efficiency (a): (baseline: 10%), P/B ratio: (baseline: 30), Trophic level (n): (baseline: 2) and Energy conversion efficiency (E): (baseline: 2.5 kJ/g). The results are shown in Table 2.

**TABLE 2:** Sensitivity analysis of fish potential production and fish carrying capacity Index to parameter variation.

Parameter	Variation range	FPP RMSE (%)	FCCI > 0.6 Probability (%)	Key driver
Trophic efficiency (*a*)	8%–12%	±9.7	1.2	Moderate
P/B ratio	24%–36%	**±12.4**	**1.8**	**Primary**
Trophic level (*n*)	1.6–2.4	±2.1	0.3	Minor
Energy conversion (*E*)	2.0–3.0 kJ/g	±1.9	0.1	Minor

A Monte Carlo simulation was conducted, four key parameters were varied using uniform distributions across ±20% of baseline values. Convergence was assessed by running iterative batches (*n* = 500, 800, 1000); the coefficient of variation for FPP stabilised after 800 iterations, confirming that 1000 iterations were sufficient. The mean FPP across runs varied by < 0.5% between the final 200 iterations. The resulting FPP and FCCI distributions were analysed to compute RMSE and the probability of exceeding the sustainability threshold (FCCI > 0.6). The results demonstrated that the variability in FPP was predominantly driven by changes in the P/B ratio (RMSE = ±12.4%), while having minimal impact (RMSE = ±1.9%) due to its energy conversion for species‐specific stability. Importantly, the FCCI values remained below the 0.6 sustainability threshold in > 98% of the simulations across all 23 years (Table 3), aligning with the observed long‐term trends. Spatial heterogeneity in FPP (Figure 6) further buffered parameter uncertainty, as cyclical climate‐driven fluctuations exceeded biological parameter noise. The trophic level (*n* = 2) exhibited negligible sensitivity (RMSE = ±2.1%), confirming its invariance for the naked carp's stenophagous diet. These findings illustrate the model's robustness for Qinghai Lake's simplified ecosystem, where species dominance minimises trophic plasticity.

**TABLE 3:** Gross Annual FPP, FP and Lake‐wide Mean FCCI for each year from 2002 to 2024.

Year	FP (tonnes)	FPP (tonnes)	FCCI
2002	2592	173,957	0.0149
2003	3610	184,870	0.0195
2004	5017	130,983	0.0383
2005	6926	309,105	0.0224
2006	15,274	245,807	0.0621
2007	20,176	142,251	0.1418
2008	24,340	129,996	0.1872
2009	27,260	105,769	0.2577
2010	30,120	197,943	0.1522
2011	32,069	180,573	0.1776
2012	33,569	233,566	0.1437
2013	39,005	154,401	0.2526
2014	50,500	225,917	0.2235
2015	62,100	381,049	0.1630
2016	70,800	205,590	0.3444
2017	81,200	272,255	0.2983
2018	88,000	260,456	0.3379
2019	93,000	328,986	0.2827
2020	93,000	240,334	0.3870
2021	108,500	244,526	0.4437
2022	114,100	264,900	0.4307
2023	120,300	206,540	0.5825
2024	127,500	244,581	0.5213

## 4 Discussion

### 4.1 Uncertainty in FPP Estimation and Comparison With Other Studies

**FIGURE 10:**
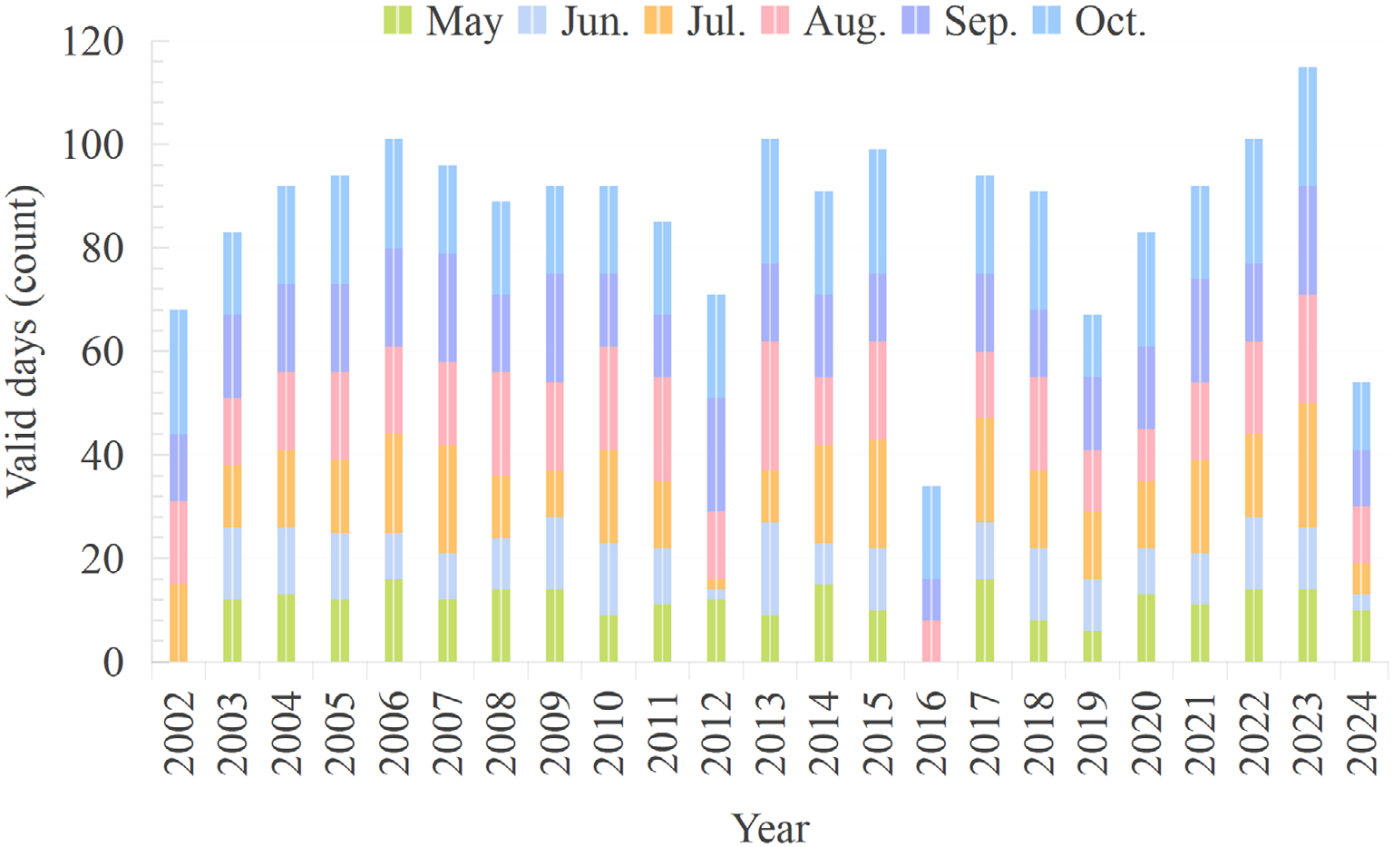
Statistics for the number of effective days of chlorophyll *a* in OC data products during each month from 2002 to 2024.

Estimating FPP in large, alpine lakes is inherently subject to uncertainties arising from methodological choices, data limitations and ecosystem complexity. A primary source of uncertainty lies in the satellite‐derived inputs, particularly Chl‐a retrievals. In optically complex waters like Qinghai Lake, factors such as coloured dissolved organic matter from glacial‐fed tributaries, high salinity and turbidity from monsoon runoff can lead to Chl‐a overestimation by standard ocean colour algorithms (Li et al. 2016; Wei et al. 2022; Xu et al. 2017). Although our in situ regression correction mitigated this error (ARE = 10.71%), residual uncertainties persist, especially in productive estuary zones where they disproportionately affect local FPP estimates (Figure 6). Furthermore, the uneven temporal distribution of valid satellite data (Figure 10), with higher availability in late summer and autumn, required careful gap‐filling to avoid biasing monthly and seasonal FPP trends.

The choice of PP model introduces another layer of uncertainty. Our evaluation demonstrated that the VGPM outperformed alternatives like the CbPM and the Eppley‐VGPM Hybrid for Qinghai Lake's specific conditions. The CbPM's reliance on particulate backscattering proved unreliable due to glacial flour in tributary estuaries, while the Eppley‐VGPM hybrid overestimated PP by neglecting phosphorus limitation in oligotrophic waters (Li et al. 2020; Zhou et al. 2017). Therefore, VGPM, with its explicit joint parameterisation of temperature and Chl‐a, currently provides the most reliable PP estimates for this system, though future cross‐validation with independent sensors (e.g., Sentinel‐3 OLCI) across diverse plateau lakes is warranted to fully assess model portability (Toté et al. 2024).

Beyond remote sensing, assumptions in the ecological energy transfer model contribute to FPP uncertainty. Key parameters include the trophic transfer efficiency (*a* = 10%), the production‐to‐biomass ratio (P/B = 30) and the energy conversion factor (*E* = 2.5 kJ/g) (Yao et al. 2011). However, the remarkable simplicity and stability of Qinghai Lake's ecosystem—dominated (> 95% biomass) by the stenophagous naked carp with a fixed trophic level (*n* = 2)—buffer against significant biological variability (Weng et al. 2023). These parameters, derived from and validated by long‐term local studies, exhibit minimal seasonal or interannual fluctuation (Cai et al. 2023; Zhang et al. 2013). Our Monte Carlo sensitivity analysis confirmed that while the P/B ratio was the most influential parameter (RMSE = ±12.4%), the probability of FCCI exceeding sustainable thresholds remained low (< 2%) across all simulations, underscoring the model's robustness for this mono‐species system (Table 2).

When compared with previous estimates, our satellite‐driven FPP values for Qinghai Lake (42,000–176,000 t annually) are significantly higher than the 26,760 t obtained from traditional field surveys limited to 2006–2010 (Yao et al. 2011). This discrepancy highlights the principal advantage of our framework: the ability to provide spatially explicit, lake‐wide and long‐term (2002–2024) estimates that overcome the logistical and spatial limitations of infrequent point sampling. Placing our results in a global context reveals both similarities and distinctions. The peak FPP in Qinghai Lake is comparable to annual estimates for large oligotrophic systems like Lake Superior (Harvey and Kitchell 2000). However, the pronounced seasonality and strong year cyclical fluctuations observed in Qinghai Lake (Figure 7) reflect the heightened sensitivity of plateau lakes to climatic drivers, contrasting with the more moderate seasonal variation in eutrophic lowland lakes like Lake Taihu (Deng et al. 2017). The spatial heterogeneity of fishery pressure, indicated by FCCI hotspots in the northwest (Figure 8), mirrors patterns observed in marine systems like the Baltic Sea (Rosenberg et al. 2014), but the overall FCCI remaining below 0.6 suggests Qinghai Lake's fishery potential is currently underutilised, unlike many overexploited global fisheries.

### 4.2 Fish Carrying Capacity Index and Implications for Sustainable Fisheries Management

To develop an effective fisheries management strategy for Qinghai Lake, we analysed the relationship between naked carp fish production, releasing stocked fish populations and estimated FPP from 2002 to 2024 using FCCI. In order to ensure that biomass did not exceed food availability, it was necessary to maintain a FCCI no greater than 1.0 (Knight and Jiang 2009). If a FCCI is greater than 1, naked carp exhibits a 12% reduction in mean body length (from 32.4 cm to 28.5 cm) due to food competition, as observed in a sample of 1200 specimens from northwest high‐pressure zones (FCCI > 1) versus southeast underutilised areas (FCCI < 0.3; see Figure 1, Sites B). This aligns with the findings of Yao et al. (2011), who reported that zooplankton scarcity at high densities reduced growth rates by 0.3 cm/year.

It is desirable to maintain FCCI at ≤ 1.0 to ensure that fish biomass does not exceed the food energy supplied by PP. A FCCI is greater than 1.0 would indicate unsustainable exploitation, whereas the consistently observed FCCI is less than 0.6 (Figure 9) reflects the current underutilisation of available resources. In regions of the lake where FCCI exceeds 1, such as the northwestern area. The pronounced northwestern FCCI hotspots align with two key ecological processes: (1) the seasonal zooplankton maxima driven by nutrient inputs from the Buha and Quanji Rivers, which attract high densities of filter‐feeding naked carp; and (2) the primary spawning migration routes that funnel fish into these same estuarine zones from May to September. Consequently, the high FCCI in the northwest reflects both high fish aggregation and elevated local food demand. Therefore, we recommend optimising restocking efforts and conducting regular monitoring to reduce competition and ensure sustainability. In contrast, in the southeastern region of the lake with lower nutrient inputs and weaker migratory attraction, where FCCI is below 1, fishery resources (Behrenfeld and Falkowski 1997) are underutilised, potentially alleviating density‐dependent competition in the northwest while leveraging available production capacity in the southeast, thereby supporting a more sustainable fishery management strategy.

While biotic factors such as predation, competition and disease theoretically influence the carrying capacity, Qinghai Lake's simplified trophic structure and long‐term conservation measures minimise these impacts. Naked carp comprise more than 95% of the fish biomass, eliminating significant interspecific competition. No other fish species exceeds 5% biomass, as confirmed by 23 years of gill‐net and echo sounder surveys (Shi et al. 2016). Aquatic predation is negligible due to the absence of piscivorous fish (Cai et al. 2023). Bird predation (e.g., by *Larus ichthyaetus* ) accounts for less than 0.5% of annual FP, a loss that is offset by restocking programmes (O'Bryan et al. 2010; Tian et al. 2019).

Strict prohibition of fishing since 2002 has removed anthropogenic mortality, allowing the ecosystem to stabilise around the population dynamics of naked carp. While no disease outbreaks were observed during 2002–2024, high‐altitude stressors (e.g., rapid temperature shifts Guo and Tang 2021; Li et al. 2016) could heighten susceptibility. The stability of the FCCI (Figure 9) suggests current resilience, but proactive monitoring for pathogens (e.g., *Aeromonas*) is recommended, especially in high‐density northwest zones (FCCI > 0.6). The robustness of the FCCI (consistently < 0.6) reflects the effective absence of biotic pressures in this managed system. However, disease‐induced mortality could locally elevate FCCI by reducing FP without altering FPP.

### 4.3 Future Research Directions

The present study suggests several future research directions. First, refining the estimation methods for FPP and FCCI is essential. This includes determining more accurate food chain energy transfer efficiency parameters through biological experiments and integrating multi‐source data, such as satellite remote sensing, drone monitoring, and in situ surveys, to improve spatial and temporal resolution. Specifically, drone and autonomous monitoring systems offer transformative potential for high‐altitude lakes such as Qinghai. A feasible implementation strategy would deploy seasonal drone campaigns during August–October (coinciding with peak FPP and minimal ice cover), as validated by Chl‐a data availability and solar‐powered uncrewed surface vehicles. This tiered approach leverages drones for high‐resolution calibration while relying on satellites for broad‐scale FPP mapping, overcoming alpine logistics constraints (Wang et al. 2023).

Second, establishing a long‐term ecological monitoring network in and around Qinghai Lake is essential. Regular monitoring of key parameters, such as the water temperature, chlorophyll *a*, primary productivity and fish resources, will validate and enhance modelling results. Finally, exploring sustainable fishery utilisation models through ecosystem‐based management strategies and interdisciplinary research in ecology, fisheries science, environmental science and economics will provide comprehensive scientific support for sustainable fishery management in Qinghai Lake. Such an assessment would help to evaluate fishery resources and forecast FPP for restocking and sustainable fisheries management, also providing a benchmark for future assessments in large plateau lakes.

Looking forward, integrating climate‐sensitive covariates into the FPP and FCCI framework could enhance its predictive capacity. Satellite‐derived indicators such as summer surface temperature anomalies, ice cover duration and interannual PP variability are key drivers of FPP in alpine lakes. Coupling these with downscaled climate projections would allow scenario‐based forecasting of fishery carrying capacity under future warming regimes, transforming the present diagnostic tool into a proactive planning asset for climate‐resilient fishery management.

The present results can be generalised to other endemic, cold‐water systems. The FCCI framework is transferable wherever four key conditions are met: (i) dominance of a single endemic fish species (> 80% of total biomass), (ii) availability of ≥ 20‐year MODIS or equivalent chlorophyll *a* archived data, (iii) documented trophic level and energy conversion parameters and (iv) at least periodic acoustic or mark‐recapture estimates of fish biomass. African Rift lakes, South American altiplano lakes and Arctic oligotrophic lakes all satisfy these criteria (Cohen et al. 2000; Danilov and Ekelund 2001; De Los Rios and Bayly 2018; Ekdahl et al. 2008; Olaka et al. 2010; Svenning and Borgstrom 2024). Pilot tests in these regions would merely require re‐parameterising Equation (1) for the local trophic transfer efficiency (a), the P/B ratio and the energy conversion factor (E), followed by cross‐validation against existing fishery‐independent surveys. Thus, FCCI offers a scalable tool for data‐limited, high‐altitude and high‐latitude ecosystems worldwide.

## Author Contributions

**Ban Xuan:** conceptualization (lead), data curation (lead), formal analysis (lead), funding acquisition (equal), investigation (equal), methodology (equal), project administration (equal), resources (equal), software (equal), supervision (equal), validation (equal), visualization (equal), writing – original draft (equal), writing – review and editing (equal). **Qi Hongfang:** conceptualization (equal), data curation (equal), funding acquisition (lead), investigation (lead), project administration (lead), writing – review and editing (equal). **Shu Peng:** data curation (equal), formal analysis (equal), methodology (equal), software (equal), validation (equal), visualization (equal). **Luo Ying:** data curation (equal), investigation (equal), methodology (equal). **Ling Feng:** conceptualization (equal), methodology (equal), writing – review and editing (equal). **Xiao Fei:** conceptualization (equal), methodology (equal). **Li Pengchen:** methodology (equal). **Fu Shengyun:** investigation (equal). **Du Hao:** conceptualization (equal), investigation (equal), writing – review and editing (equal).

## Funding

This work was supported by Qinghai Province Science and Technology Achievement Transformation Special Project (grant number 2024‐SF‐152).

## Conflicts of Interest

The authors declare no conflicts of interest.

## Supporting information


**Appendix S1:** ece372989‐sup‐0001‐AppendixS1.docx.

**Appendix S2:** ece372989‐sup‐0002‐AppendixS2.docx.

**Tables S1–S5:** ece372989‐sup‐0003‐TablesS1‐S5.docx.

## Data Availability

The satellite product data for temperature, Chlorophyll a, Photosynthetically Active Radiation and diffuse attenuation coefficient at 490 nm download from the websites https://oceancolor.gsfc.nasa.gov/; the daily photoperiod data applied from the websites http://data.cma.cn/. The field data is in the support information.
