# Supplementary material for: Satellite‐Driven Synthesis of Fish Production Dynamics and Carrying Capacity Mechanisms in a High‐Altitude Lake Ecosystem
Source: Ecol Evol. 2026 Feb 17;16(2):e72989. doi: 10.1002/ece3.72989 (PMC12912843; doi:10.1002/ece3.72989)
Supplement: Supplementary file 1 — Appendix S1: ece372989‐sup‐0001‐AppendixS1.docx. [file ECE3-16-e72989-s001.docx]

Python code for calculation phytoplankton primary production(PP) by using Vertically Generalised Production Model (VGPM)

import sys

import arcpy

import numpy

from pandas import read_excel

from datetime import datetime, timedelta

from os.path import split, exists

from os import makedirs, getcwd

def get_coe(filename):

correct_coe = dict()

correct_coe["A"] = dict()

correct_coe["T"] = dict()

correct_coe["A"]["month"] = []

correct_coe["A"]["a"] = []

correct_coe["A"]["b"] = []

correct_coe["T"]["month"] = []

correct_coe["T"]["a"] = []

correct_coe["T"]["b"] = []

with open(filename, "r") as ff:

line = ff.readline()

line = ff.readline()

while line:

items = line.replace("\n", "").split("\t")

correct_coe[items[1]]["month"].append(int(items[0]))

correct_coe[items[1]]["a"].append(float(items[2]))

correct_coe[items[1]]["b"].append(float(items[3]))

line = ff.readline()

return correct_coe

if __name__ == '__main__':

arcpy.env.overwriteOutput = True

arcpy.CheckOutExtension('spatial')

years = range(2002, 2025)

cell_size = 1500

correct_coe = get_coe(ur"dataa/coe.txt")

dirr_df = read_excel(getcwd() + "\\data\\mon.xlsx")

for year in years:

# region Fp Calculation

workspace = unicode(file_path + '\\' + 'result.gdb')

arcpy.env.workspace = workspace

try:

arcpy.CreateFileGDB_management(file_path + '\\', 'result.gdb')

except:

pass

result_path = getcwd() + r"\\data\\result\\" + str(year) + "\\"

for month in correct_coe[A_T]["month"]:

# region File Input

sst_file = file_path + '\\sst\\rasters\\mean_rasters.gdb\\sst_' + str(year) + '_' + str(month)

chlor_a_file = file_path + '\\chlor_a\\rasters\\mean_rasters.gdb\\corrected_chlor_a_' + str(year) \

+ '_' + str(month)

Kd_490_file = file_path + '\\Kd_490\\rasters\\mean_rasters.gdb\\Kd_490_' + str(year) + '_' + str(month)

par_file = file_path + '\\par\\rasters\\mean_rasters.gdb\\par_' + str(year) + '_' + str(month)

Dirr = dirr_df[["Hours", "Month"]][dirr_df["Year"] == year]

Dirr = float(Dirr["Hours"][Dirr["Month"] == month]) / day_moths[month]

T = arcpy.sa.Raster(sst_file)

chlor_a = arcpy.sa.Raster(chlor_a_file)

E0 = arcpy.sa.Raster(par_file)

Kd_490 = arcpy.sa.Raster(Kd_490_file)

# endregion

Pept = arcpy.sa.Con(T <= -1.0, 1.13, arcpy.sa.Con(T >= 28.5, 4.0, 1.2956 + 2.749 * 0.1 * T +

6.17 * 0.01 * T * T - 2.05 * 0.01 * T ** 3 + 2.462 * 0.001 * T ** 4 -

1.348 * 0.0001 * T ** 5 + 3.4132 * 0.000001 * T ** 6 - 3.27 * 0.1 ** 8 * T ** 7))

Pept.save('Pept_' + str(year) + "_" + str(month))

Zeu = 4.605 / 0.896 / Kd_490 ** 0.873

PPeu = 0.66125 * Pept * E0 / (E0 + 4.1) * Zeu * chlor_a * Dirr

Fp = PPeu * 0.10 ** 2 * 30 / 2.5

Fp.save(result_path + "Fp_" + str(year) + "_" + str(month) + ".tif")

PPeu.save(result_path + "PPeu_" + str(year) + "_" + str(month) + ".tif")

chlor_a.save(result_path + "Chlor_a_" + str(year) + "_" + str(month) + ".tif")
