# Supplementary material for: Satellite‐Driven Synthesis of Fish Production Dynamics and Carrying Capacity Mechanisms in a High‐Altitude Lake Ecosystem
Source: Ecol Evol. 2026 Feb 17;16(2):e72989. doi: 10.1002/ece3.72989 (PMC12912843; doi:10.1002/ece3.72989)
Supplement: Supplementary file 2 — Appendix S2: ece372989‐sup‐0002‐AppendixS2.docx. [file ECE3-16-e72989-s002.docx]

**SUPPORTING INFORMATION**

S1. Calculation of Primary Production via the VGPM model

The PP in the surface water layer was calculated using the VGPM model based on the satellite data. A brief note of the VGPM equation and parameters adopted in this study is provided in Table S3 (Behrenfeld & Falkowski, 1997; Gaoping & Liangqiang, 2004).

PP = 0.66125 * $P_{\mathrm{ept}}^{B}$ * $\frac{E_{0}}{E_{0}+4.1}$ * $Z_{\mathrm{eu}}$ * $C_{\mathrm{opt}}$ * $D_{\mathrm{irr}}$ (S1)

Otherwise

$P_{ept}^{B}$ = $\left\{ \begin{aligned} \begin{matrix} 1.13 (T\leq-1.0) \\ 4.00 (T\geq28.5) \\ P_{ept}^{B} (-1.0<T<28.5) \end{matrix} \\ \end{aligned} \right\}$ (S2)

$P_{ept}^{B}=1.2956+2.749\times{10}^{-1}\times T+6.17\times{10}^{-2}\times T^{2}-2.05\times{10}^{-2}\times T^{3}+2.462\times{10}^{-3}\times T^{4}-1.348\times{10}^{-4}\times T^{5}+3.4132\times{10}^{-6}\times T^{6}-3.27\times{10}^{-8}\times T^{7}$ (S3)

$Z_{eu}$=$4.605/{Kd}_{\left( \mathrm{PAR} \right)}$ (S4)

Kd_(PAR)_ =0.896Kd_(490)_^0.873^, (r_2_ = 0.98, n = 81, p<0.001), (S5)

where PP is the primary productivity (mg C/m^2^/day) integrated from the surface layer to the euphotic layer; $P_{\mathrm{ept}}^{B}$ is the maximum rate of carbon fixation in the water column (mg C/mg Chl·h); $E_{0}$ is the photosynthetically available radiation (PAR) at the water surface (μmol/m^2^/day); $Z_{\mathrm{eu}}$ is the depth of the true photosphere (m); $D_{\mathrm{irr}}$ is the photoperiod (h); and $C_{\mathrm{opt}}$ is the chlorophyll *a* concentration (Chl-a) (µg/L) at the water surface.

S2. Calculation of Fish Production

Fish density and spatial distribution were quantified using a BioSonics DT-X echosounder (EY60 model, 210 kHz split-beam transducer, 6.8° beam width) in conjunction with biological catch statistics. Given the confirmed individual distribution and low-density aggregation of naked carp (*Gymnocypris przewalskii*), the Single Target Strength Analysis (STSA) method was applied:

1. Survey Design: 12 north-south transects (5 km spacing) covered all depth zones across 4,500 km^2^, with data acquired at 5–6 knots vessel speed and 5 pings/sec.
2. Target Detection: Individual fish targets were identified from acoustic backscatter (Sv > −50 dB) after noise removal (−70 dB threshold). The TS values were calculated from the fish length (L) of naked carp via species-specific regression in Qinghai Lake (Love & Richard, 1977):

$TS=19.1\times\log_{10}\left( L \right)-0.9\times\log_{10}\left( f \right)-$62, (S6)

where TS represents the target strength value (dB); L represents the target body length (cm); f represents the frequency of the echo sounder transducer (kHz), which was 210 kHz in this study.

This survey considered thousands of pings as one sampling unit in each survey. The average fish density of the entire lake area was obtained according to the density of each sampling unit, and the quantity of naked carp in Qinghai Lake was estimated in combination with the water volume of the lake.

$\rho_{i}=\frac{S_{i}\times1000}{V_{i}}$ (S7)

$V_{i}=\sum_{i=1}^{3000} V_{p}$ (S8)

$V_{p}=\frac{1}{3}\pi\tan\left( \frac{\theta}{2} \right)\left( H^{3}-h \right)$ (S9)

$\rho_{a}=\frac{1}{n}\sum_{i=1}^{n} \rho_{i}$ (S10)

$N=\rho_{a}\times V_{Q}$ (S11)

$B=\alpha\times N\times W_{a}$, (S12)

where $\text{ρ}_{\text{i}}\text{ }$represents the fish density of the *i*_th_ detection unit (ind./1000 m^3^); $\text{S}_{\text{i}}$ represents the number of individuals identified in the *i*_th_ detection unit; $\text{V}_{\text{i}}\text{ }$represents the volume of water in the *i*_th_ detection unit; $\text{V}_{\text{p}}$ represents the volume of water detected by each ping; $\text{θ}$ represents the beam angle of the sonar; H represents the depth detected by each ping; h represents the detection blind zone (near field) of the echo sounder itself, which is 1 m; $\text{ρ}_{\text{a}}$ represents the average density of naked carps in the entire area of Qinghai Lake area; N represents the total number of naked carp in Qinghai Lake. $\text{V}_{\text{Q}}$ represents the total volume of Qinghai Lake; $\text{W}_{\text{a}}$ represents the average weight of naked carp; B represents the exploitable resource quantity of naked carp in Qinghai Lake, and $\alpha$ represents the driving coefficient.

1. Biomass Calculation:

Lake-wide fish production (FP) was derived by estimating the fish density from target counts per sampled volume. This was scaled to the total lake volume using echo integration (5-m-depth bins) and converted to biomass via length-weight relationships from concurrent gill-net catches (50 sites per year).

1. Validation:

Methodological accuracy was validated through mark-recapture studies with 500 tagged individuals per year (2018–2024), yielding a mean biomass estimation error of ±7.2% and confirming the reliability of this approach for long-term monitoring in the lake's unique high-altitude ecosystem.

**Table S1. The information of the sampling data in the surface water of Qinghai Lake.**

| **Month** | **Date** | **Time** | **Number of Samples** | **Chlorophyl-a (µg·L^−1^)** | **Surface Water Temperature (°C)** | | **Average Density of Phytoplankton**  **(ind·L^−1^)** | **Biomass of Phytoplankton (mg·L^−1^)** |
| --- | --- | --- | --- | --- | --- | --- | --- | --- |
|  |  |  |  | **Range** | **Average** | **Average** | **Average** | **Average** |
| May | 14-15 May 2018 | 7:30~19:50 | 12 | 0.030~0.245 | 0.089 | 8.8 | 2400 | 0.0093 |
| July | 1-2 July 2018 | 8:00~19:38 | 20 | 0.070~0.788 | 0.404 | 15 | 21,400 | 0.0823 |
| August | 7-10 August 2018 | 8:10~19:15 | 29 | 0.070~0.730 | 0.258 | 17.5 | 50,730 | 0.238 |
| September | 26-27 September 2018 | 6:40~19:02 | 19 | 0.020~1.229 | 0.42 | 13.4 | 39,470 | 0.126 |
| August | 2-5 August 2019 | 6:30~18:30 | 30 | 0.134~0.594 | 0.295 | 13 | 49,268 | 0.221 |
| September | 19-20 September 2019 | 7:20~17:50 | 24 | 0.196~0.605 | 0.364 | 10 | 38,456 | 0.119 |
| June | 30 June – 2 July 2021 | 8:45~16:20 | 25 | 0.198~0.475 | 0.296 | 11 | 14,399 | 0.044 |
| August | 19-23 August 2021 | 8:50~17:50 | 24 | 0.034~0.536 | 0.266 | 15 | 56,687 | 0.154 |
| June | 23-24 June 2022 | 7:06~17:16 | 23 | 0.211~0.719 | 0.354 | 15.5 | 44,106 | 0.673 |
| May | 25-26 May 2023 | 7:10~17:13 | 22 | 0.04~1.642 | 0.258 | 8.4 | 42,061 | 0.695 |
| August | 10-13 August 2023 | 7:10~18:30 | 29 | 0.14~3.23 | 1.404 | 17.4 | 52,693 | 1.857 |

**Table S2. Details of the field and satellite data sources used in this model**

| **NO** | **Data** | **Fielddata** | **Temporalandspatialresolution** | **Online/satellitedata** |
| --- | --- | --- | --- | --- |
| 1 | Sea surface temperature  (T) | Field survey | 1km*1km  Daily | NASA/GSFC MODIS data processing center  <https://oceancolor.gsfc.nasa.gov/> |
| 2 | Chlorophyl-a  (Chl-a) | laboratory experiment | 1km*1km  Daily | NASA/GSFC MODIS data processing center  <https://oceancolor.gsfc.nasa.gov/> |
| 3 | Diffuse Attenuation Coefficient for 490 wavelengths (Kd_490_) |  | 1km*1km  Daily | NASA/PO.DAAC AVHRR oceans pathfinder  <https://oceancolor.gsfc.nasa.gov/> |
| 4 | Photosynthetically Active Radiation (PAR) |  | 1km*1km  Daily | NASA/PO.DAAC AVHRR oceans pathfinder  <https://oceancolor.gsfc.nasa.gov/> |
| 5 | Density of phytoplankton | laboratory experiment |  |  |
| 6 | Phytoplankton Primary Production (PP) | Field survey |  |  |
| 7 | Fish density and fish weight | Field survey |  |  |
| 8 | Daily photoperiod |  | monthly | China Meteorological Data Service Center  <http://data.cma.cn/> |

**Table S3. The parameters used in the VGPM model and estimation of fish potential production**

| **Symbol** | **Description** | **Units** |
| --- | --- | --- |
| PP | Daily Phytoplankton Primary Productivity from the surface to euphotic depth | mg C/m^2^/day |
| P^B^_ept_ | Optimal rate of daily carbon fixation within a water column | mg C/ (mg Chl•h) |
| E_0_ | Daily photosynthetically active radiation (PAR) | mol quanta/m^2^ |
| Z_eu_ | Physical depth (m) of the euphotic zone defined as the penetration depth of 1 % surface irradiance | m |
| D_irr_ | Daily photoperiod | h |
| Chl-a | Chlorophyll-a concentration | µg/L |
| T | Sea Surface Temperature | ℃ |
| Kd _(PAR)_ | Diffuse Attenuation Coefficient for photosynthetically active radiation (PAR) | m^-1^ |
| Kd_(490)_ | Diffuse Attenuation Coefficient for 490 wavelengths | m^-1^ |
| FPP | Fish potential production | g/m^2^/day |

**Table S4 The error statistics between the measured and modeled parameters**

| **Parameters** | **CC** | **ARE** | **RMSE** | **EA** |
| --- | --- | --- | --- | --- |
| **Rectified Chl-a** | 0.79 | -10.71% | 0.07 | 72.29% |
| **T products** | 0.88 | -1.77% | 1.08 | 92.64% |
| **PP** | 0.95 | 26.70% | 11.61 | 75.97% |

Note: CC is the correlation coefficient; ARE is the average relative error; RMSE is the root mean square error; EA is the accuracy of the estimation.

**Table S5. Unit conversion framework for derived FPP from primary production**

| **Step** | **Parameter** | **Input Units** | **Conversion Factor** | **Output Units** | **Biological Basis** |
| --- | --- | --- | --- | --- | --- |
| 1 | Daily Net PP | mg C m⁻² day⁻¹ | × days/month | mg C m⁻² month⁻¹ | Temporal integration |
| 2 | Trophic transfer | mg C m⁻² month⁻¹ | × (0.1)² | mg C m⁻² month⁻¹ | 10% transfer per trophic level (n=2) |
| 3 | Production scaling | mg C m⁻² month⁻¹ | × 30 | mg C m⁻² month⁻¹ | P/B ratio for naked carp |
| 4 | Energy conversion | mg C m⁻² month⁻¹ | ÷ 2.5 | g fresh weight m⁻² month⁻¹ | 2.5 kJ/g fish wet weight; 1 kJ ≡ 1 mg C |
