# Supplementary material for: Satellite‐Driven Synthesis of Fish Production Dynamics and Carrying Capacity Mechanisms in a High‐Altitude Lake Ecosystem
Source: Ecol Evol. 2026 Feb 17;16(2):e72989. doi: 10.1002/ece3.72989 (PMC12912843; doi:10.1002/ece3.72989)
Supplement: Supplementary file 3 — Tables S1–S5: ece372989‐sup‐0003‐TablesS1‐S5.docx. [file ECE3-16-e72989-s003.docx]

**Table S1. The information of the sampling data in the surface water of Qinghai Lake.**

| Month | Date | Time | Number of Samples | Chlorophyl-a (µg·L^−1^) | Surface Water Temperature (°C) | | Average Density of Phytoplankton  (ind·L^−1^) | Biomass of Phytoplankton (mg·L^−1^) |
| --- | --- | --- | --- | --- | --- | --- | --- | --- |
|  |  |  |  | **Range** | **Average** | **Average** | **Average** | **Average** |
| May | 14-15 May 2018 | 7:30~19:50 | 12 | 0.030~0.245 | 0.089 | 8.8 | 2400 | 0.0093 |
| July | 1-2 July 2018 | 8:00~19:38 | 20 | 0.070~0.788 | 0.404 | 15 | 21,400 | 0.0823 |
| August | 7-10 August 2018 | 8:10~19:15 | 29 | 0.070~0.730 | 0.258 | 17.5 | 50,730 | 0.238 |
| September | 26-27 September 2018 | 6:40~19:02 | 19 | 0.020~1.229 | 0.42 | 13.4 | 39,470 | 0.126 |
| August | 2-5 August 2019 | 6:30~18:30 | 30 | 0.134~0.594 | 0.295 | 13 | 49,268 | 0.221 |
| September | 19-20 September 2019 | 7:20~17:50 | 24 | 0.196~0.605 | 0.364 | 10 | 38,456 | 0.119 |
| June | 30 June – 2 July 2021 | 8:45~16:20 | 25 | 0.198~0.475 | 0.296 | 11 | 14,399 | 0.044 |
| August | 19-23 August 2021 | 8:50~17:50 | 24 | 0.034~0.536 | 0.266 | 15 | 56,687 | 0.154 |
| June | 23-24 June 2022 | 7:06~17:16 | 23 | 0.211~0.719 | 0.354 | 15.5 | 44,106 | 0.673 |
| May | 25-26 May 2023 | 7:10~17:13 | 22 | 0.04~1.642 | 0.258 | 8.4 | 42,061 | 0.695 |
| August | 10-13 August 2023 | 7:10~18:30 | 29 | 0.14~3.23 | 1.404 | 17.4 | 52,693 | 1.857 |

**Table S2. Details of the field and satellite data sources used in this model**

| **NO** | **Data** | **Fielddata** | **Temporalandspatialresolution** | **Online/satellitedata** |
| --- | --- | --- | --- | --- |
| 1 | Sea surface temperature  (T) | Field survey | 1km*1km  Daily | NASA/GSFC MODIS data processing center  <https://oceancolor.gsfc.nasa.gov/> |
| 2 | Chlorophyl-a  (Chl-a) | laboratory experiment | 1km*1km  Daily | NASA/GSFC MODIS data processing center  <https://oceancolor.gsfc.nasa.gov/> |
| 3 | Diffuse Attenuation Coefficient for 490 wavelengths (Kd_490_) |  | 1km*1km  Daily | NASA/PO.DAAC AVHRR oceans pathfinder  <https://oceancolor.gsfc.nasa.gov/> |
| 4 | Photosynthetically Active Radiation (PAR) |  | 1km*1km  Daily | NASA/PO.DAAC AVHRR oceans pathfinder  <https://oceancolor.gsfc.nasa.gov/> |
| 5 | Density of phytoplankton | laboratory experiment |  |  |
| 6 | Phytoplankton Primary Production (PP) | Field survey |  |  |
| 7 | Fish density and fish weight | Field survey |  |  |
| 8 | Daily photoperiod |  | monthly | China Meteorological Data Service Center  <http://data.cma.cn/> |

**Table S3. The parameters used in the VGPM model and estimation of fish potential production**

| **Symbol** | **Description** | **Units** |
| --- | --- | --- |
| PP | Daily Phytoplankton Primary Productivity from the surface to euphotic depth | mg C/m^2^/day |
| P^B^_ept_ | Optimal rate of daily carbon fixation within a water column | mg C/ (mg Chl•h) |
| E_0_ | Daily photosynthetically active radiation (PAR) | mol quanta/m^2^ |
| Z_eu_ | Physical depth (m) of the euphotic zone defined as the penetration depth of 1 % surface irradiance | m |
| D_irr_ | Daily photoperiod | h |
| Chl-a | Chlorophyll-a concentration | µg/L |
| T | Sea Surface Temperature | ℃ |
| Kd _(PAR)_ | Diffuse Attenuation Coefficient for photosynthetically active radiation (PAR) | m^-1^ |
| Kd_(490)_ | Diffuse Attenuation Coefficient for 490 wavelengths | m^-1^ |
| FPP | Fish potential production | g/m^2^/day |

**Table S4 The error statistics between the measured and modeled parameters**

| Parameters | CC | ARE | RMSE | EA |
| --- | --- | --- | --- | --- |
| Rectified Chl-a | 0.79 | -10.71% | 0.07 | 72.29% |
| T products | 0.88 | -1.77% | 1.08 | 92.64% |
| PP | 0.95 | 26.70% | 11.61 | 75.97% |

Note: CC is the correlation coefficient; ARE is the average relative error; RMSE is the root mean square error; EA is the accuracy of the estimation.

**Table S5. Unit conversion framework for derived FPP from primary production**

| **Step** | **Parameter** | **Input Units** | **Conversion Factor** | **Output Units** | **Biological Basis** |
| --- | --- | --- | --- | --- | --- |
| 1 | Daily Net PP | mg C m⁻² day⁻¹ | × days/month | mg C m⁻² month⁻¹ | Temporal integration |
| 2 | Trophic transfer | mg C m⁻² month⁻¹ | × (0.1)² | mg C m⁻² month⁻¹ | 10% transfer per trophic level (n=2) |
| 3 | Production scaling | mg C m⁻² month⁻¹ | × 30 | mg C m⁻² month⁻¹ | P/B ratio for naked carp |
| 4 | Energy conversion | mg C m⁻² month⁻¹ | ÷ 2.5 | g fresh weight m⁻² month⁻¹ | 2.5 kJ/g fish wet weight; 1 kJ ≡ 1 mg C |
